# Variation in the genomic locations and sequence conservation of STAR elements among staphylococcal species provides insight into DNA repeat evolution

**DOI:** 10.1186/1471-2164-13-515

**Published:** 2012-09-28

**Authors:** Joanne Purves, Matthew Blades, Yasrab Arafat, Salman A Malik, Christopher D Bayliss, Julie A Morrissey

**Affiliations:** 1 Department of Genetics, University of Leicester, University Road, Leicester LE1 7RH, UK; 2 Bioinformatics and Biostatistics Analysis Support Hub (B/BASH), The Centre for Core Biotechnology Services, University of Leicester, University Road, Leicester LE1 7RH, UK; 3 Department of Biochemistry, Quaid-i-Azam University, Islamabad, 45320, Pakistan

## Abstract

**Background:**

*Staphylococcus aureus* Repeat (STAR) elements are a type of interspersed intergenic direct repeat. In this study the conservation and variation in these elements was explored by bioinformatic analyses of published staphylococcal genome sequences and through sequencing of specific STAR element loci from a large set of *S*. *aureus* isolates.

**Results:**

Using bioinformatic analyses, we found that the STAR elements were located in different genomic loci within each staphylococcal species. There was no correlation between the number of STAR elements in each genome and the evolutionary relatedness of staphylococcal species, however higher levels of repeats were observed in both *S*. *aureus* and *S*. *lugdunensis* compared to other staphylococcal species. Unexpectedly, sequencing of the internal spacer sequences of individual repeat elements from multiple isolates showed conservation at the sequence level within deep evolutionary lineages of *S*. *aureus*. Whilst individual STAR element loci were demonstrated to expand and contract, the sequences associated with each locus were stable and distinct from one another.

**Conclusions:**

The high degree of lineage and locus-specific conservation of these intergenic repeat regions suggests that STAR elements are maintained due to selective or molecular forces with some of these elements having an important role in cell physiology. The high prevalence in two of the more virulent staphylococcal species is indicative of a potential role for STAR elements in pathogenesis.

## Background

*Staphylococcus aureus* repeat (STAR) elements are short GC rich direct repeats found in intergenic regions across the *S*. *aureus* genome [1]. STAR elements consist of 14 bp direct repeats of the consensus sequence T(G/A/T)TGTTG(G/T)GGCCC(C/A) interspersed with at least 40 bp of recurring sequences [1]. The function, origin and the mechanism by which STAR elements propagate throughout staphylococcal genomes is unknown.

Repetitive DNA sequences are ubiquitous in eukaryotic and prokaryotic genomes, and are highly diverse in their structure and function. While eukaryotic repeat elements often have no clear role within the cell, prokaryotic repeat elements tend to be functionally significant [2,3]. These roles include transcriptional or translational phase variation of gene expression [4], modulation of mRNA transcript stability [5] and in the case of the well characterised CRISPR elements protecting the genome from invading foreign DNA elements [6]. Currently no function has been described for STAR elements.

Repetitive elements can evolve rapidly over time. For simple sequence repeats, such as homopolymeric tracts, slip-strand mispairing during DNA replication can result in a change in repeat number after a single generation. This is the basis of phase variable gene regulation, providing random switching of target genes between ON and OFF states and resulting in bacterial subpopulations that are better adapted to environmental change [4,7]. Mutations in tandem repeats, resulting in changes in repeat number, occur 100–10,000 times more frequently than point mutations making repeat arrays hotspots for genomic plasticity [8]. Interspersed repeats can undergo homologous recombination, resulting in changes in repeat number and the spread of a repeat element throughout the genome [9]. Therefore genomic repeats are inherently unstable and can undergo dramatic changes over time, which may or may not be linked to their function.

Since their initial discovery over a decade ago there has been little published data regarding STAR elements, and much of what has been published has focused on their potential as variable number tandem repeats (VNTR) and their use in *S*. *aureus* strain typing [10,11]. Information regarding the abundance and conservation of these repeat elements throughout the *Staphylococcus* genus stem from techniques such as Southern blotting that do not provide resolution to the sequence level [1,10], and a study using comparative genomics which identified some copy number variation in a truncated STAR element (TGTTGNGGCCC) between a select subset of *S*. *aureus* strains [12]. Current advances in genome sequencing have meant that there is now a wealth of available staphylococcal genome sequences, allowing us to study the structure and evolution of STAR elements in much finer detail. The purpose of this work was to analyse STAR elements at the molecular level from both a wide variety of *S*. *aureus* strains and in other staphylococcal species in order to further our understanding of the origin, propagation and maintenance of this repeat element.

Through the use of whole genome pattern searches we have extensively mapped the locations of STAR elements in 15 *S*. *aureus* genomes as well as 7 staphylococcal species, alongside a more detailed look at individual STAR loci from a wider pool of *S*. *aureus* strains at the sequence level. The data show that STAR elements are associated with distinct flanking genes in each staphylococcal species, suggesting that they are maintained autonomously within each species, and that their positions within each genome are stable over time. Furthermore *S*. *aureus* STAR elements are conserved at the sequence level within ancient evolutionary lineages. These features point towards an as yet unidentified function for these repeat elements.

## Results

### STAR elements are significantly more abundant in both repeat number and genomic location in *S*. *aureus* and *S*. *lugdunensis* compared with other staphylococci

Although STAR elements have previously been shown to be much more abundant in *S*. *aureus* genomes than those of other staphylococcal species [1], the techniques employed only provided semi-quantitative data on the actual numbers of repeat motifs involved and did not give any indication of the exact number of elements present in each genome or into how many distinct loci these fall.

The available *S*. *aureus* and staphylococcal genomes were probed *in silico* for the presence of the degenerate STAR repeat sequence TNTGTTGNGGCCC using the RSA genome-scale pattern-search tool (http://rsat.ulb.ac.be/). The above sequence was chosen to provide enough degeneracy to identify all “true” STAR elements conforming to the original description from Cramton *et al*. [1], and as used in the MVLA schemes [13-15] while limiting the identification of spurious STAR elements. We hypothesised that the abundance of STAR elements in staphylococcal species other than *S*. *aureus* would vary depending on the relatedness of that species to *S*. *aureus*, with more closely related species containing similar numbers of elements. Based on 16S rDNA sequence comparison [16,17], *S*. *aureus* is most closely related to *S*. *epidermidis*, followed by *S*. *haemolyticus*, then *S*. *lugdunensis*, *S*. *saprophyticus*, *S*. *pseudintermedius* and finally *S*. *carnosus*.

In each *S*. *aureus* strain examined, between 62 and 90 STAR motifs were found, occurring at 32 to 39 distinct locations in each genome (referred to as STAR loci) (Table 1). The number of motifs at a particular locus varied between strains; the majority of loci contain only a single repeat motif however some tracts contain as many as 7. Unexpectedly *S*. *lugdunensis* contains a similar abundance of STAR motifs to *S*. *aureus*, with 72 identified at 39 loci, while the more closely related *S*. *epidermidis* and *S*. *haemolyticus* contain far fewer than *S*. *aureus*. *S*. *epidermidis* ATCC1228 contains 17 motifs at 8 different loci, while *S*. *epidermidis* RP62A contains 19 motifs at 7 different loci and *S*. *haemolyticus* contains 3 STAR motifs each at individual loci. *S*. *pseudintermedius*, *S*. *saprophyticus* and *S*. *carnosus* are all devoid of STAR motifs. The prevalence of these repeats is not, therefore, correlated with the phylogenetic relationships of the species, suggesting that the high levels of STAR motifs found in *S*. *aureus* and *S*. *lugdunensis* are due to other selective or molecular forces.

**Table 1:** Abundance of individual STAR element motifs and STAR element loci in different staphylococcal genomes

**Species**	**Strain**	**Number of STAR motifs**	**Number of STAR loci**
*S*. *aureus*	ED98	77	33
*S*. *aureus*	RF122	63	35
*S*. *aureus*	COL	78	34
*S*. *aureus*	JH1	74	33
*S*. *aureus*	JH9	74	36
*S*. *aureus*	MRSA252	62	32
*S*. *aureus*	MSSA476	84	39
*S*. *aureus*	MW2	90	39
*S*. *aureus*	Mu3	80	34
*S*. *aureus*	Mu50	80	34
*S*. *aureus*	N315	81	34
*S*. *aureus*	NCTC8325	77	32
*S*. *aureus*	USA300 FRP3757	74	34
*S*. *aureus*	USA300 TCH1516	75	34
*S*. *aureus*	Newman	83	34
*S*. *carnosus*	TM300	0	N/A
*S*. *epidermidis*	ATCC12228	17	8
*S*. *epidermidis*	RP62A	19	7
*S*. *haemolyticus*	JCSC1435	3	3
*S*. *lugdunensis*	HKU09-01	72	39
*S*. *pseudintermedius*	ED99	0	N/A
*S*. *pseudintermedius*	HKU10-03	0	N/A
*S*. *saprophyticus*	ATCC 15305	0	N/A

STAR element pattern searches were performed with an increased motif degeneracy of one additional substitution allowed throughout the sequence. Although additional, weaker STAR motifs were identified in each species tested, the increase in motif number was proportional to the number of “true” elements present so that *S*. *aureus* and *S*. *lugdunenesis* still showed a higher prevalence of STAR motifs compared with other staphylococcal species (data not shown).

### STAR elements locations are conserved within *S*. *aureus*, but not between different staphylococcal species

In order to provide insight into the evolution of STAR elements as species and strains diverged over time, the conservation of the positions of STAR loci between and within staphylococcal genomes was determined. A total of 72 potential STAR loci were identified for *S*. *aureus*, with each strain containing between 32 and 39 loci (Table 2 & Additional file 1: Table S3). Strains from the same evolutionary lineage carry the same STAR loci, and therefore the STAR elements have not disseminated to new genome positions since the lineages diverged from one another. This indicates that the elements are stable within the *S*. *aureus* genome.

**Table 2:** **Locations and conservation of STAR element in 15 *S***. ***aureus *genomes**

***S. aureus *genomes**															
**Locus No**.	**A**	**B**	**C**	**D**	**E**	**F**	**G**	**H**	**I**	**J**	**K**	**L**	**M**	**N**	**O**
**1**			1												
**2**	2	2	1	2	1	1	1	1	1	1	1	2	2	2	1
**3**			1												
**4**			1	2	1	1	1	1	1	1					
**5**	3	3									1	1	1	1	1
**6**			1	1											
**7**											1	1	1	1	1
**8**	2	2	3	2											
**9**				1											
**10**	2	2	2	1	2	2	2	2	2	2	4	4	4	4	4
**11**					2	2	1	2	2	2					
**12**	1	1	4		1	1	1	1	1	1	1	1	1	1	1
**13**			1								1	1	1	1	1
**14**			1	3											
**15**	1	1	1		1	1	1	1	1	1	1	1	1	1	1
**16**			2	2*	3	3	2	3	3	3					
**17**				1											
**18**				1											
**19**	1	1	3	1	2	2	2	2	2	2	2	2	2	2	2
**20**	3	3		2	1	1	1	1	1	1	2	2	4	3	3
**21**	3	3	5	1	2	2	3	3	3	3	3	3	3	3	3
**22**	1	1	1								1		1	1	1
**23**				2											
**24**	1	1		1	1	1	3	3	3	3	7	6	4	4	7
**25**			1												
**26**				2											
**27**	3	3	2	1	2	2		2	2	2	1	1	1	1	1
**28**	3	3	1	1	4	4	4	4	5	4					
**29**	1	1			2	2	2	2	2	2					
**30**	2	3	1	2	2	2	2	2	2	2	2	2	2	2	2
**31**	1	2													
**32**	3	3			3	3	1	1	1	1	2	2	2	2	2
**33**			1												
**34**	1	1			1	1	1	1	1	1	1	1	1	1	1
**35**	3	2			4	4	4	4	4	4	3	3	3	3	3
**36**				1											
**37**				2											
**38**	2	4		2											
**39**	1	1			1	1	1	1	1	1	1	1	1	1	1
**40**			2												
**41**	2	4		1	3	3	3	3	3	3	4	2	2	2	4
**42**	4	4			4	4	5	4	4	4	5	5	2	2	5
**43**	2	2	2								2	2	2	2	2
**44**	2	2	4	3	2	2	2	2	2	2	2	2	2	2	2
**45**			1												
**46**	2	2	1	2	2	2	2	1	1	1	3	3	3	3	3
**47**			3												
**48**	4	4	2**	2**	4	4	4	4	4	4	4	4	4	4	4
**49**	2	1			3	3	4	6	6	6	3	1	1	3	3
**50**	3	3	3	4	5	5	3	6	6	6	2	7	5	5	5
51	2	2	1	1	3	3	3	3	3	3	2	3	3	3	3
**52**				1											
**53**			2												
**54**	4	4		2							4	4	4	4	4
**55**	3	3	2	2	4	4	4	3	3	4	3	2	2	2	3
**56**					1	1	1	1	1	1	1	1	1	1	1
**57**					2	2	2	2	2	2					
**58**				1											
**59**				1											
**60**	1	1									1	1	1	1	1
**61**	1	1													
**62**	1	1	1	1	3	3	3	3	3	3	4	3	4	4	4
**63**			2												
**64**	3	4	1**	1			2	2	2	2	1		1	1	1
**65**	4	4													
**66**	2	2			1	1	3	2	2	2					
**67**					1	1	1	1	1	1					
**68**				1											
**69**	2	2									2	2	2	2	2
**70**				2											
**71**				2											
**Total loci**	**39**	**39**	**32**	**36**	**33**	**33**	**33**	**34**	**34**	**34**	**34**	**32**	**34**	**34**	**34**

The presence and number of STAR motifs at each potential STAR locus identified from each of the following *S*. *aureus* genomes: (A) MSSA476, (B) MW2, (C) MRSA252, (D) RF122, (E) JH1, (F) JH9, (G) ED98, (H), Mu3, (I)Mu50, (J) N315, (K) COL, (L) NCTC8325, (M) USA300 FRP3757, (N) USA300 TCH1516, (O) Newman. * indicates only the upstream gene matches. ** indicates only the downstream gene matches. Annotations for unknown genes are taken from MSSA476, MRSA 252 and RF122. Full details of adjacent loci are available in Table S3 (Additional file 1).

The *S*. *aureus* STAR reference set was then used to extend this analysis to the additional staphylococcal genomes, in order to determine whether the STAR elements are associated with particular genes across different species. Homologues to several of the *S*. *aureus* flanking regions in the reference set were identified across the staphylococcal species, but none of these alignments contained STAR elements.

Reference sets for both *S*. *epidermidis* and *S*. *haemolyticus*, were then used to determine STAR locus conservation between *S*. *epidermidis*, *S*. *haemolyticus* and *S*. *lugdunensis*. We did not find a single STAR associated genomic neighbourhood that was consistent between two species, although the STAR associated loci were conserved between the two *S*. *epidermidis* genomes studied. These data show that STAR elements have spread through and been maintained autonomously within each staphylococcal genome.

### The *gapR* STAR locus differs in structure between strains but contains consistent regions of sequence variability

In order to determine how an individual STAR locus can alter as isolates diverge from one another, and therefore draw conclusions about how these repeat elements evolve over time, a single STAR locus was selected and analysed at the sequence level from a diverse pool of *S*. *aureus* strains. The STAR locus found upstream of the highly conserved *S*. *aureus* glycolytic operon, which is essential for glucose metabolism [18], was selected as this STAR locus showed high variability in the number of motifs between strains in our initial study. The intergenic region between *gapR* and the upstream open reading frame was sequenced from a total of 37 *S*. *aureus* isolates from a range of sources (See additional file 1: Table S1). The sequence of this region was also extracted from the 15 sequenced *S*. *aureus* genomes described above, providing data for a total of 52 *S*. *aureus* strains.

**Figure 1:**
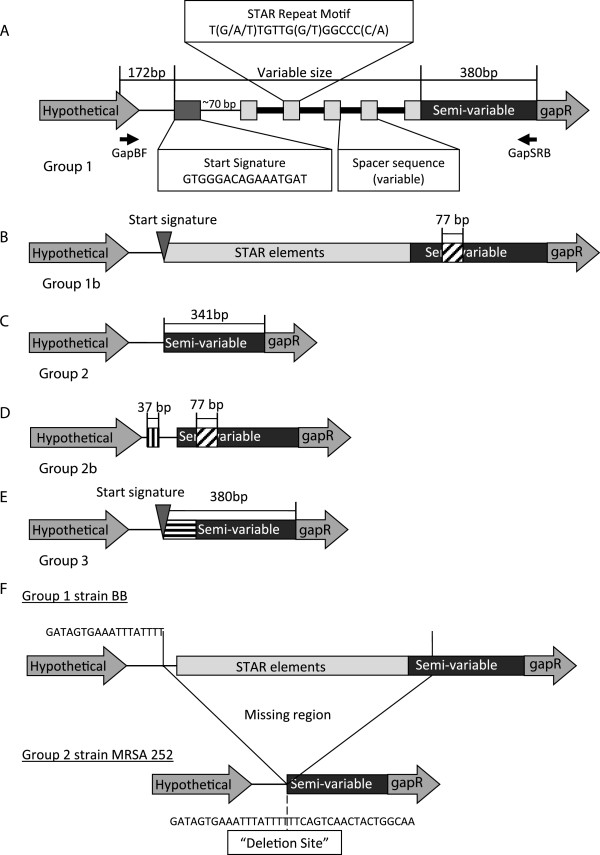
**Schematic representations of the Group 1 (A), Group 1b (B), Group 2 (C), Group 2b (D), and Group 3 (E) structural groups showing variation in the region upstream of *gapR*. **(**A**) Primer positions and important conserved sequence motifs are indicated. Identical 77 bp insertions within the semi-variable region (diagonal cross-hatch) were identified in Groups 1b and 2b. Group 2b contains an unrelated 37 bp insertion upstream of the STAR deletion site (vertical cross-hatch). Group 3 contains the STAR start signature followed by 70 bp of sequence unrelated to other STAR elements or semi-variable regions examined (horizontal cross-hatch). (**F**) Schematic representation of the *gapR* STAR element deletion site, comparing the locus from strain BB and MRSA252 and indicating the region missing from the Group 2 strains. The conserved sequences flanking the deletion site are highlighted in each strain.

Comparison of the DNA sequence of the *gapR* STAR locus between *S*. *aureus* strains revealed a large amount of variability in this region, including differences in both repeat number and large scale structural changes (Figure 1A). In the majority of strains (33/52) the *gapR* STAR locus begins with a “start signature” sequence of GTGGGACAGAAATGAT, which is slightly truncated compared to the sequence initially identified at the *hprK* STAR locus [1]. This is followed by between 1 and 6 conserved STAR motifs interspersed with 40-44 bp of “spacer” sequence, which shows some variability between strains. Between the STAR elements and the *gapR* coding region there is a 380 bp “semi-variable” region, which shares approximately 88% sequence identity between strains. This is classified as the Group 1 structure.

In 9 of the strains examined (Group 2) the entire STAR element locus is missing, as well as the first 39 bp of the 5' end of the semi-variable region (Figure 1C). All of the Group 2 strains identified share 100% sequence identity across the sequenced region. An alternative deletion event appears to have resulted in the Group 3 structure (in 5/52 strains), which retains the STAR start signature but shows no evidence of any STAR element repeat sequences (Figure 1E). In addition, the first 70 bp of the semi-variable region in this group shares little similarity with the semi-variable region or the STAR element sequences identified in any other strains.

The final two structural variants, Groups 1b and 2b, appear to be derivatives of Group 1 and 2 respectively. Group 2b is missing the STAR elements having the same precise deletion site as Group 2. Both Group 1b and Group 2b have an identical 77 bp insertion within the semi-variable region (Figure 1B), whilst Group 2b has a second 37 bp insertion 27 bp upstream of the STAR element deletion site (368 bp upstream of ATG) (Figure 1D). The 37 bp insertion seen in Group 2b does not share any sequence similarity with the 77 bp insertion.

### STAR element structural Groups 2 and 3 are restricted to specific evolutionary lineages

**Figure 2:**
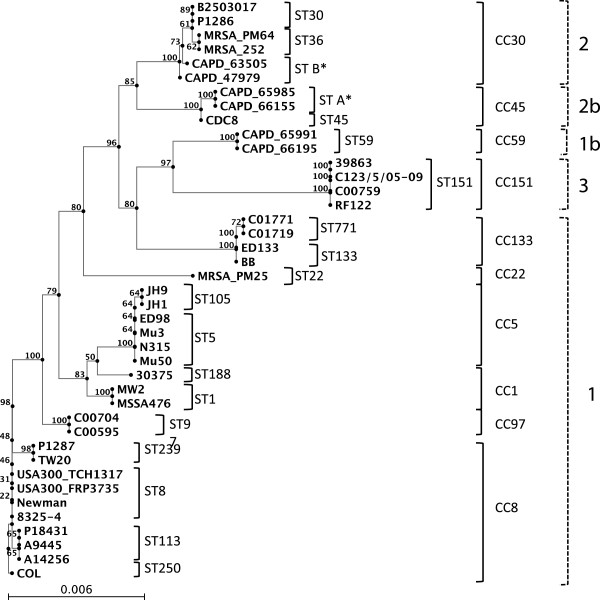
**The Neighbour-joining tree was derived from the concatenated MLST profiles of each of the *S. aureus *strains examined in this study, based on pairwise multiple alignment (ClustalW). **The *gapR* STAR locus structural group of each of the strains is also highlighted, indicating how the structural groups cluster into specific clades.

Multi locus sequence typing (MLST) was used to investigate whether identify the different STAR element structural groups were associated with particular evolutionary lineages of *S*. *aureus*. ST types were derived for each of the strains and then a phylogenetic tree was derived using the Neighbour-joining algorithm based on the MLST profiles to determine the evolutionary relationships between these strains (Figure 2).

These ST-based phylogenetic trees indicated that the Group 2 and Group 3 strains, which do not contain STAR elements at the *gapR* locus, fall into distinct evolutionary lineages compared to the Group 1 strains (Figure 2). All of the Group 2 strains (ST30, ST36, ST34, novel ST B), which are 100% conserved across the *gapR* STAR locus, fall into clonal complex (CC) 30 (Figure 2). As all of the CC30 strains examined in this study have a Group 2 structure, loss of STAR elements in these strains most probably occurred in a common ancestor and was maintained as the ST’s diverged from one another. Interestingly all of the Group 3 strains, which have a partial loss of the STAR element locus, belong to ST 151 (CC 151). As the entire sequenced region is 100% conserved between the ST151 strains, this again suggests that the deletion occurred early in the evolution of this sequence type and has been maintained in subsequent isolates.

Surprisingly the Group 1b and Group 2b strains, which contain the same unique sequence insertion, fall into distinct clonal complexes with very different allelic profiles; the Group 1b strains are from ST59 (CC 59) and the Group 2b strains are from ST45 and novel ST A, which are both in CC45. Although it initially appeared that the Group 1b and Group 2b structures were derived from Group 1 and Group 2, the phylogenetic data indicates that this is not a recent event. Furthermore these structures did not occur due to a recent loss/gain of the STAR locus between Groups 1b and 2b, as these strains are from different CC’s. Taken together these data suggest that the *gapR* STAR locus differences occurred in very early lineages of *S*. *aureus* and have been maintained at a level equal to that of CC in subsequent strains.

### Sequence variation of the STAR element spacers correlates with evolutionary lineage

**Figure 3:**
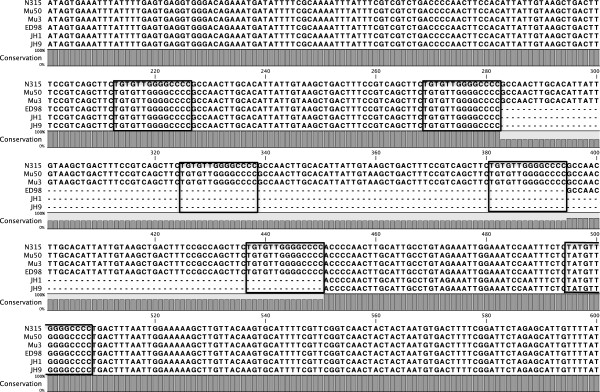
**Alignment of the *gapR *STAR locus from CC5. **Each STAR motif is highlighted.

**Figure 4:**
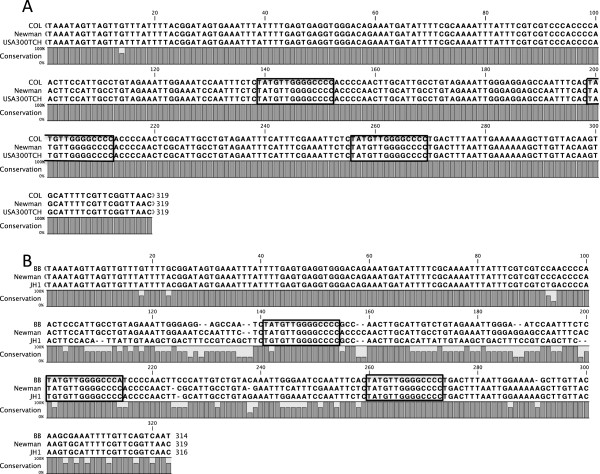
**Alignment of the 3 STAR element motifs at the *gapR *STAR locus from (A) three related strains from ST8 (COL, Newman and USA300 TCH1516) and (B) 3 unrelated strains (BB, Newman and JH1). **STAR motifs are highlighted.

As the Groups 1 strains fall into a wide range of ST’s and CC’s, it is clear that *gapR* STAR locus structure alone does not correlate with any particular lineage. However analysis of this STAR locus at the sequence level shows that the sequences of the “spacers”, which occur between STAR motifs, are strongly conserved within CC’s. For example, in strains from CC5 and CC8 the STAR spacer sequences are 100% identical between isolates even though the number of repeat motifs varies (Figure 3, Figure 4A). Interestingly the final spacer sequence (between the final and penultimate STAR element) is distinct from the internal spacers, but this “anchor” spacer is again 100% conserved between strains of the same lineage. In contrast, alignment of the spacers from strains originating from different lineages, even where they contain the same repeat number, detected high levels of variation in these sequences between distinct CC’s (Figure 4B). We have confirmed the conservation of spacer sequences within a CC’s in all strains tested here, with the exception of the two strains representing CC97. The spacer sequences from the CC97 strains C00595 and C00704 are still highly conserved, but they are not 100% identical. This is further evidence that the structure and sequence of the *gapR* STAR locus is maintained within distinct evolutionary lineages.

### STAR spacer sequences are distinct at different loci within *S*. *aureus* strains but still correlate with lineage

**Figure 5:**
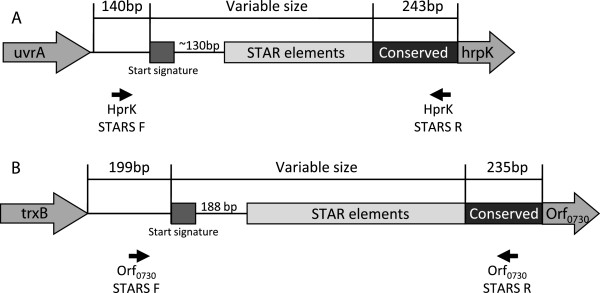
**Schematic representations of (A) the structure of the *hprK *STAR element locus including the position of primers HprK F and HprK R and (B) the structure of the *orf***_***0730 ***_**STAR element locus including the position of primers Orf**_**0730 **_**F and Orf**_**0730 **_**R**.

Two additional STAR loci were analysed to further investigate the link between STAR element conservation and evolutionary lineage. The STAR loci found upstream of both the *hprK* gene, encoding a Hpr kinase/phosphorylase, and a gene of unknown function SAS0730, referred to as *orf*_*0730*_ in this study, were chosen as RSAT analysis of these regions shows that they both contained variable numbers of STAR motifs and are preceded by a start signature. The STAR element regions upstream of *hprK* and *orf*_*0730*_ were either PCR amplified and sequenced from a selection of *S*. *aureus* strains using primer pairs HprK F + HprK R and Orf_0730_ F + Orf_0730_ R respectively (Figure 5) or extracted from the 15 complete genome sequences. The strains were chosen to include at least 2 examples, where possible, of strains from each lineage identified previously (see Table 3).

**Table 3:** **STAR element repeat units at the *hrpK *and *orf***_***0730 ***_**loci**

**Strain**	**MLST sequence type**	**No**. **of *gapR *STAR element repeats**	**No**. **of *hprK *STAR element repeats**	**No**. **of *orf***_***0730 ***_**STAR element repeats**
BB	133	3	1	5
C01719	771	3	1	5
C01771	771	3	1	4
30375	188	4	1	3
66195	59	4	2	3
65991	59	4	2	3
C00704	97	1	1	4
C00595	97	2	1	3
RF122	151	0	1	5
C00759	151	0	1	4
C123/5/05-09	151	0	1	6
8325-4	8	1	3	7
Newman	8	3	3	5
USA300 TCH1516	8	3	3	5
USA300 FRP3735	8	1	3	5
Mu50	5	6	3	6
N315	5	6	3	6
Mu3	5	6	3	6
ED98	5	4	3	3
MSSA476	1	2	2	3
Mw2	1	1	2	3
JH1	105	3	3	5
JH9	105	3	3	5
COL	250	3	2	2
MRSA252	36	0	2	3
TW20	239	3	2	4

**Figure 6:**
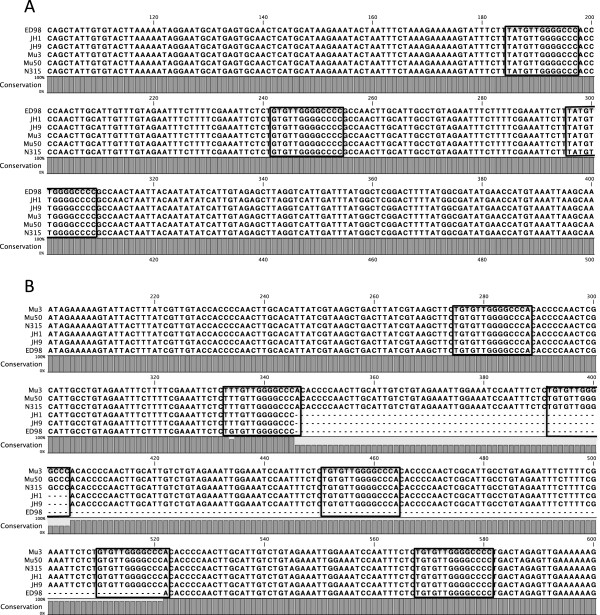
**Alignments of sequencing from (A) the *hprK *STAR locus and (B) the *orf***_***0730***_**STAR locus from strains belonging to CC5.** The STAR motifs are highlighted in each case.

Interestingly both the *hprK* and *orf*_*0730*_ STAR loci have some key structural differences to that of the *gapR* STAR locus. The STAR start signature sequence is present at both loci but occurs ~130 bp and 188 bp upstream of the first repeat motif at the *hprK* and *orf*_*0730*_ loci respectively, compared to ~70 bp at the *gapR* STAR locus (Figure 5). Furthermore there is no evidence for different structural variants in any of the strains examined as both the *hprK* and *orf*_*0730*_ STAR elements only follow the Group 1 STAR element structure found at the *gapR* locus. There is also less variability in the number of STAR element repeat motifs at each of these loci, with the *hprK* locus ranging from 1–3 repeats and the *orf*_*0730*_ locus ranging from 3–7, compared with the 1–6 repeats seen at the *gapR* locus. Sequence analysis of the *hprK* and *orf*_*0730*_ STAR spacers showed that sequence level variation in these repeat regions still strongly correlates with lineage as seen at the *gapR* locus. Alignments of each individual locus clearly demonstrate high levels of conservation of the spacer sequences within strains from a particular lineage (data not shown), as shown for CC5 (Figure 6). For strains containing multiple STAR repeats at locus *orf*_*0730*_, we observed two distinct spacer types within the same locus in some strains, as seen in CC5 (Figure 6). However it is important to note that these sequences are still 100% conserved within each lineage and do not occur at either the *hprK* or *gapR* loci in any of the strains examined supporting the observation that the spacer sequences are distinct from one another and that there is no frequent transfer of motifs/spacers between the STAR loci.

## Discussion

In this study we have taken advantage of the wealth of fully annotated staphylococcal genomes to take a detailed look at STAR elements. To our knowledge this is the first in depth study of these interspersed repeats at the sequence level across multiple staphylococcal species, providing a unique insight into their evolution.

STAR elements are highly abundant in *S*. *aureus* and yet we have shown that strain variation in the STAR element nucleotide sequences strongly correlates with their evolutionary lineage, as derived by MLST. This is unexpected as intergenic regions such as the STAR loci, which consist of repetitive elements dispersed throughout the genome, would be expected to show a high level of mutation and hence evolve at a higher rate than the conserved functional MLST loci where mutations are observed at a very low rate [19]. These findings suggest STAR elements are functional and may be under strong purifying selection.

STAR elements were sequenced from the *gapR*, *hprK* and *orf*_*0730*_ loci from multiple *S*. *aureus* strains. In the majority of loci where multiple STAR repeats were present, the spacer sequences were often identical or differed by 1–3 nucleotides resulting in tandem repeats of ~50 nucleotides. These repetitive sequences should be unstable and exhibit frequent alterations in repeat number due to slip-strand mispairing during DNA replication. This process is likely to drive rapid alterations in repeat number, but not sequence, at many of these loci, as found with some other bacterial tandem repeats [3,20,21]. Congruent with this theory, strains belonging to the same ST contain identical or highly conserved spacer sequences between the interspersed STAR motifs at a specific locus even when repeat numbers varied. This also suggests that localised expansion and contraction of the repeat region occurs even as the strains diverge from one another.

In contrast, the spacer sequences are distinct at each STAR locus, even within a particular genome. Due to the repetitive nature of STAR elements it has previously been suggested that homologous recombination between repeats occurs as a means of large scale genomic rearrangements [1], or could provide a simple means of propagating these repeats at different loci throughout the genome. As the spacers are distinct between unrelated strains and at different STAR loci within a strain, homologous recombination is unlikely to be occurring at a high frequency between STAR loci either intergenomically or intragenomically. Either of these processes would result in gene conversion and the emergence of a dominant spacer sequence variant across multiple loci, a phenomenon we did not identify in this study. From the evidence presented here we suggest that the process of varying repeat number within a locus is limited to duplication or deletion of motifs from within that locus during DNA replication or repair and is not due to recombination with elements present elsewhere in the genome. We also suggest that the mechanism for dispersal of the STAR elements to new positions throughout the *S*. *aureus* genome may not involve recombination as originally hypothesised.

The *gapR* STAR locus was the least structurally stable of the three loci studied. The loss of the elements in the Group 2 and 2b structure occurs at the same “deletion” site and the surrounding DNA is undisturbed compared to that of the Group 1 and 1b strains. This is similar to another class of interspersed bacterial repeats known as Enterobacterial repetitive intergenic consensus (ERIC) sequences, which have been identified across the eubacterial kingdom [22]. The sequence surrounding an inserted ERIC remains unchanged, indicating a precise insertion or deletion event via a mechanism distinct from classic transposition mechanisms [23,24]. It is unclear whether a similar conserved mechanism is involved in the total loss or gain of STAR loci or whether the deletion site is merely acting as a hotspot for STAR element translocation. The partial loss of elements seen in strains such as RF122 (Group 3) does not occur at this deletion site, and may represent a different mechanism of repeat propagation or an error in repeat translocation in an ancestral strain that has been maintained in subsequent generations. There is no evidence of the total loss or gain of the *gapR* STAR locus in the recent evolution of *S*. *aureus* strains, as both the Group 2 and Group 3 isolates fall into distinct evolutionarily lineages. This strongly implies that the deletion process is infrequent and that the loss or gain of the *gapR* STAR locus may have occurred in early ancestors of these lineages and been retained in subsequent isolates. Pourcel *et al*. observed a similar complex structure for the STAR elements in the SA0906 locus (locus 28 in this study) with restriction of specific structural variants to certain lineages [11]. These findings provide further evidence of the conservation of each of the STAR loci within a strain and lineage.

Our observed correlation between evolutionary lineage and both the structure of the *gapR* locus and the spacer sequences of the *gapR*, *hprK* and *orf*_*0730*_ loci, suggests that STAR element loci retain lineage-specific phylogenetic information and may be utilised as major determinants of lineage in typing schemes. The genome wide mapping of STAR elements across the 15 *S*. *aureus* strains studied here identified 12 loci that were present in every genome sequence and a further 11 loci that were present in 85% of the genome sequences. The vast majority of these loci (20/23) contain more than one repeat and exhibit variable repeat numbers (data not shown), making them prime candidates for the development of future typing schemes. Some STAR loci have already been utilised in typing schemes for *S*. *aureus*, first using an RFLP typing method [10], and more recently as part of a greater *m*ultiple-*l*ocus *v*ariable-number tandem-repeat analysis (MLVA) scheme alongside other variable-number tandem repeats (VNTR’s) and staphylococcal interspersed repeat units (SIRU) [11,13-15]. The recent extended MLVA scheme utilised six STAR element loci of which five were completely conserved in a collection of 240 strains [11], although only four are present in up to 85% of the strains studied here. Therefore our highly conserved loci should be examined for their potential value as markers of lineages.

We have found that the STAR elements are not restricted to specific genomic neighbourhoods across staphylococcal species. This would suggest that the elements are not simply decaying from some early Staphylococcus progenitor as this genus has diverged over time, but rather that each species has acquired STAR elements as independent events, which have then undergone proliferation to distinct locations in each genome. Furthermore, STAR elements are maintained at a much higher level in the *S*. *aureus* and *S*. *lugdunensis* genomes compared to other staphylococcal species. The higher prevalence of these elements in *S*. *aureus* and *S*. *lugdunensis* may be due to the presence of a dispersal mechanism (e.g. a transposase mechanism) that is absent in the other species studied here, the absence of a mechanism to prevent spread of repetitive elements in these two species or strong selection for the function of these elements.

The highly conserved nature of STAR elements within a CC suggests a functional role. Unlike eukaryotic genomes which can contain more than 50% repetitive DNA [2], prokaryotic genomes are streamlined as the propagation of non-functional “selfish” DNA is a burden to the rapidly dividing organisms and selected against [3,25]. Other repeat elements in bacteria have functions in cell physiology, such as transcriptional control [5] and protection of the microbial genome against foreign DNA [6,26,27]. A functional role for STAR elements is supported by evidence showing that some STAR elements are present in the leader regions of mRNAs although the significance of this for gene expression has yet to be investigated further [28]. Alternatively, these repetitive sequences may have a general function in chromosome structure or stability, as seen with some eukaryotic repeat elements [29], which has led to their maintenance and spread within staphylococcal genomes. The STAR repeats are found associated with loci encoding virulence factors, metal transporters and several essential metabolic enzymes. The significance of the STAR repeats in the intergenic regions of these particular loci requires further investigation.

Interestingly, both *S*. *aureus* and *S*. *lugdunensis* tend to be much more pathogenic in humans compared to other staphylococcal species [30] with *S*. *lugdunensis* N920143 having several homologues of *S*. *aureus* virulence and colonisation factors that are not found in other staphylococcal species [31]. Our finding that STAR elements are present in higher levels in two of the more virulent staphylococcal species may indicate that the STAR elements play a role in pathogenesis. With the huge increase in the number of available genome sequences, the occurrence of STAR repeats in other bacterial species requires further investigation to confirm their existence and function outside of the staphylococcal genus.

## Conclusions

STAR elements are highly conserved at the sequence level and are maintained at high levels in both *S*. *aureus* and *S*. *lugdunensis*, but not in the other staphylococcal species studied here. Furthermore STAR elements are conserved at the sequence level within distinct evolutionary lineages but conversely exhibit localised expansion and contraction of repeats. This means that these repeat loci retain both ancient and more recent phylogenetic information, making them ideal candidates for strain typing schemes. The high level of conservation seen in these repeats suggests that STAR elements may, as with other bacterial repeats, have a functional role in cell physiology and confer fitness advantages on some or all *S*. *aureus* lineages.

## Methods

### Bacterial strains and growth conditions

A total of 41 *S*. *aureus* isolates from both human and bovine infections sources were analysed in this study (see additional file 1: Table S1). Strains were cultured in Luria Bertani medium and grown overnight at temperature of 37°C.

### Genome-wide STAR element pattern searching

The RSAT (Regulatory Sequence Analysis Tools) genome wide pattern search tool [32] was used to identify the number and location of STAR elements across the genomes of 15 *S*. *aureus* strains, 2 *Staphylococcus epidermidis* strains (ATCC12228, RP62A), 2 *Staphylococcus pseudintermedius* strains (ED99, HKU10-03), *Staphylococcus haemolyticus* (JCSC143J), *Staphylococcus lugdunensis* (HKU09-01), and *Staphylococcus saprophyticus* (ATCC 15305). The degenerate STAR element motif TNTGTTGNGGCCCN was used to identify patterns with 0 substitution on both DNA strands in each genome. The pattern search tool is available at http://rsat.ulb.ac.be/.

### STAR element locus identification and cross strain/species comparison

Using the RSAT pattern search data, each STAR locus was manually identified by determining the proximity of each STAR element to its surrounding motifs. For loci with a single element, a sequence file was extracted containing the STAR motif with 600 bp of flanking sequence either side. To prevent loci with multiple elements producing false positive matches with strings of STAR elements elsewhere in the genome, the first and last motif was extracted for each locus alongside 600 bp of upstream or downstream sequence. A reference set containing all possible *S*. *aureus* STAR loci with flanking sequences was created in FASTA format. This reference set was aligned with each complete staphylococcal genome in turn using the BLASTN algorithm with “Max Target Sequences” set to 5000. A hit table was produced containing the alignment of each reference STAR locus with its position in the target genome, % identity match and bit score. Each hit table was manually inspected to determine alignments that contained the STAR locus sequence or only the flanking sequences. The alignment data was also used to annotate the flanking genes for each STAR locus. STAR locus reference sets were also produced for *S*. *epidermidis* and *S*. *haemolyticus*, and BLASTN alignments were carried out between these reference sets and all of the other species genomes to confirm the cross species results. A reference set for *S*. *lugdunensis* was unnecessary as no matches were found with any of the other species genomes and there was only a single genome for this species.

### PCR amplification, DNA sequencing and MLST analysis

Strains were cultured in Luria Bertani broth and lysed by incubating at 37°C with lysostaphin (25ug/ml), before extraction of the genomic DNA [33]. Genomic DNA was used as a template to PCR the *gapR*, *hprK* and *orf*_*0730*_ (SAS0730) STAR element loci using appropriate primers (see additional file 1: Table S2). PCR products were purified and sequenced using the same primers. The STAR sequences were also determined *in silico* from 15 publically available *S*. *aureus* genomes (http://www.ncbi.nlm.nih.gov/). Sequences of each STAR locus were aligned using the ClustalW algorithm. Where required, MLST strain typing was carried out by PCR amplification and sequencing of internal fragments of seven MLST loci (*araC*, *aroE*, *glpF*, *gmk*, *pta*, *tpi* and *yqiL*), as described by Enright *et al*., 2000. For each strain sequence types (ST) were determined using the *S*. *aureus* MLST database (http://saureus.mlst.net/; [34]. MLST sequence types were further sorted into clonal complexes to determine common ancestry between ST’s. A Clonal Complex (CC) is defined as a group of ST’s which each has at least 5 common MLST alleles with at least one other member of the CC. A phylogenetic tree based on the MLST profiles included in this study was derived from concatemers of the 7 sequenced MLST loci fragments, using the Neighbour-joining algorithm. MLST data for all of the bovine mastitis isolates used in this study were provided by Dr. Jodi Lindsay (St. George’s University of London).

## Competing interests

The authors declare that they have no competing interests.

## Author’s contributions

JP carried out all sequence alignments, genome wide STAR element identification and annotation and molecular microbiology, participated in the design of the study and drafted the manuscript. MB carried out the cross genome STAR element comparison, and designed the methodology for this portion of the study. YA contributed to preliminary STAR element genome wide identification in *S*. *aureus*, and carried out all sequencing and typing of Pakistan MRSA isolates in this study. SAM contributed to collection and identification of the MRSA isolates from Pakistan. CDB helped with revision of the manuscript. JAM participated in the design and coordination of the study and helped with revision of the manuscript. All authors read and approve the final manuscript.

## Supplementary Material

Additional file 1 **Includes additional tables of strains and primers used in this study, and an extended version of Table **2 **identifying genes flanking each *S*. *aureus *STAR locus.** Click here for file
