# Supplementary material for: Variation in the genomic locations and sequence conservation of STAR elements among staphylococcal species provides insight into DNA repeat evolution
Source: BMC Genomics. 2012 Sep 28;13:515. doi: 10.1186/1471-2164-13-515 (PMC3532100; doi:10.1186/1471-2164-13-515)
Supplement: Additional file 1 — Includes additional tables of strains and primers used in this study, and an extended version of Table 2 identifying genes flanking each S. aureus STAR locus. [file 1471-2164-13-515-S1.docx]

**Genomic locations and sequence conservation of STAR Elements among Staphylococcal species provides insight into DNA repeat evolution**

Joanne Purves^1^, Matthew Blades^2^, Yasrab Arafat^3^, Salman A. Malik^3^, Christopher D. Bayliss^1^, Julie A. Morrissey^1^*

^1^ Department of Genetics, University of Leicester, University Road, Leicester LE1 7RH, UK.

^2^ Bioinformatics and Biostatistics Analysis Support Hub (B/BASH), The Centre for Core Biotechnology Services, University of Leicester, University Road, Leicester LE1 7RH, UK.

^3^ Department of Biochemistry, Quaid-i-Azam University, Islamabad, 45320, Pakistan.

**Table S1. *S. aureus s*trains used in this study**

| **Strain** | **Genotype/Infection source** | **Reference/Source** |
| --- | --- | --- |
| 8325-4 | NTCC8325 cured of prophages | [35] |
| Newman | Clinical MSSA isolate | [36] |
| BB | Bovine mastitis laboratory strain | [37] |
| MRSA 252 | EMRSA-16 | [38] |
| MRSA PM64 | MRSA 252 clonal variant | [39] |
| Mu50 | VISA strain | [40] |
| RF122 | Bovine mastitis associated clone | [41] |
| CDC8 | Wild type | Dr. Jodi Lindsay |
| B1203012 | Wild type / septicaemia | Queens Medical Centre, Nottingham |
| B2202016 | Wild type / septicaemia | Queens Medical Centre, Nottingham |
| B2503017 | Wild type / septicaemia | Queens Medical Centre, Nottingham |
| B0903007 | Wild type / septicaemia | Queens Medical Centre, Nottingham |
| B1003003 | Wild type / septicaemia | Queens Medical Centre, Nottingham |
| B1703012 | Wild type / septicaemia | Queens Medical Centre, Nottingham |
| SA R 4/7 | Wild type / sputum | Queens Medical Centre, Nottingham |
| SA R157/7 | Wild type / sputum | Queens Medical Centre, Nottingham |
| SA D81/7 | Wild type / wound | Queens Medical Centre, Nottingham |
| SA D196/7 | Wild type / wound | Queens Medical Centre, Nottingham |
| SA 4523-7 | Wild type / urine | Queens Medical Centre, Nottingham |
| 48064 | Wild type / CAPD* infection | NHS University Hospital’s Leicester |
| 47979 | Wild type / CAPD infection | NHS University Hospital’s Leicester |
| 63505 | Wild type / CAPD infection | NHS University Hospital’s Leicester |
| 66155 | Wild type / CAPD infection | NHS University Hospital’s Leicester |
| 65985 | Wild type / CAPD infection | NHS University Hospital’s Leicester |
| 66195 | Wild type / CAPD infection | NHS University Hospital’s Leicester |
| 65991 | Wild type / CAPD infection | NHS University Hospital’s Leicester |
| 38963 | Wild type/ bovine milk | [42] |
| 982BL | Wild type/ bovine milk | [42] |
| C00759 | Wild type/ bovine milk | [42] |
| C123/5/05-09 | Wild type/ bovine milk | [42] |
| C01865 | Wild type/ bovine milk | [42] |
| C00595 | Wild type/ bovine milk | [42] |
| C00704 | Wild type/ bovine milk | [42] |
| C01801 | Wild type/ bovine milk | [42] |
| C01719 | Wild type/ bovine milk | [42] |
| C01771 | Wild type/ bovine milk | [42] |
| A.14256 | Pakistan MRSA clinical isolate | Arafat, Malik and Bayliss, (pers. comm.) |
| A.9445 | Pakistan MRSA clinical isolate | Arafat, Malik and Bayliss, (pers. comm ) |
| P.18431 | Pakistan MRSA clinical isolate | Arafat, Malik and Bayliss, (pers. comm) |
| P.1286 | Pakistan MRSA clinical isolate | Arafat, Malik and Bayliss, (pers. comm ) |
| P.1287 | Pakistan MRSA clinical isolate | Arafat, Malik and Bayliss, (pers. comm ) |

*(CAPD = Continuous ambulatory peritoneal dialysis)

**Table S2. Primers used in this study**

| **Primer name** | **Sequence** | **Application** |
| --- | --- | --- |
| GapBF | GGGGATCCGCTAATGATAAGTAGTATTTAG | *gapR* STAR forward |
| GapSRB | GGGGATCCGTAAATAAGGATATATCACAAC | *gapR* STAR reverse |
| HprK F | CCTACTCTTACATCTCTTC | *hprK* STAR forward |
| HprK R | GTCAATCTAGAGTAGTTAAAC | *hprK* STAR reverse |
| Orf_0730_ F | CTAGAACTTAGTACGTATC | *orf_0730_* STAR forward |
| Orf_0730_ R | CATAAATCAATGTCCTAGG | *orf_0730_* STAR reverse |
| arc up | TTGATTCACCAGCGCGTATTGTC | MLST |
| arc dn | AGGTATCTGCTTCAATCAGCG | MLST |
| aro up | ATCGGAAATCCTATTTCACATTC | MLST |
| aro dn | GGTGTTGTATTAATAACGATATC | MLST |
| glp up | CTAGGAACTGCAATCTTAATCC | MLST |
| glp dn | TGGTAAAATCGCATGTCCAATTC | MLST |
| gmk up | ATCGTTTTATCGGGACCATC | MLST |
| gmk dn | TCATTAACTACAACGTAATCGTA | MLST |
| pta up | GTTAAAATCGTATTACCTGAAGG | MLST |
| pta dn | GACCCTTTTGTTGAAAAGCTTAA | MLST |
| tpi up | TCGTTCATTCTGAACGTCGTGAA | MLST |
| tpi dn | TTTGCACCTTCTAACAATTGTAC | MLST |
| yqi up | CAGCATACAGGACACCTATTGGC | MLST |
| yqi dn | CGTTGAGGAATCGATACTGGAAC | MLST |

MLST primer sequences from [31] and http://saureus.mlst.net/

**Table S3. Locations and conservation of STAR element in 15 *S. aureus* genomes**

The presence and number of STAR motifs at each given position from each *S. aureus* genome examined at each potential STAR locus identified. * indicates only the upstream gene matches. ** indicates only the downstream gene matches. Annotations for unknown genes are taken from MSSA476, MRSA 252 and RF122.

| **Locus Number** | **Strand** | **Locus orientation** | **Upstream ORF** | **Locus orientation** | **Downstream ORF** | **MSSA476** | **MW2** | **MRSA252** | **RF122** | **JH1** | **JH9** | **ED98** | **Mu3** | **Mu50** | **N315** | **COL** | **NCTC8325** | **USA300 FRP3757** | **USA300 TCH1516** | **Newman** |
| --- | --- | --- | --- | --- | --- | --- | --- | --- | --- | --- | --- | --- | --- | --- | --- | --- | --- | --- | --- | --- |
| **1** | **D** | **>** | **vraD** | **>** | **vraE** |  |  | 1 |  |  |  |  |  |  |  |  |  |  |  |  |
| **2** | **D** | **>** | **icaC** | **<** | **lipase precursor** | 2 | 2 | 1 | 2 | 1 | 1 | 1 | 1 | 1 | 1 | 1 | 2 | 2 | 2 | 1 |
| **3** | **D** | **>** | **copA copper importing ATPase A** | **>** | **SAR2639 heavy metal associated protein** |  |  | 1 |  |  |  |  |  |  |  |  |  |  |  |  |
| **4** | **D** | **<** | **SAR2519 ABC transporter ATP binding protein** | **>** | **SAR2520 putative glycerate kinase** |  |  | 1 | 2 | 1 | 1 | 1 | 1 | 1 | 1 |  |  |  |  |  |
| **5** | **D** | **>** | **Acetyltransferase (GNAT) family protein** | **<** | **SAS2416 putative Lserine dehydratase (alpha chain)** | 3 | 3 |  |  |  |  |  |  |  |  | 1 | 1 | 1 | 1 | 1 |
| **6** | **R** | **<** | **SAR2491 putative acetyltransferase** | **<** | **SAR2493 putative nitrite transporter** |  |  | 1 | 1 |  |  |  |  |  |  |  |  |  |  |  |
| **7** | **R** | **>** | **amino acid permease** | **>** | **pnbA paranitrobenzyl esterase** |  |  |  |  |  |  |  |  |  |  | 1 | 1 | 1 | 1 | 1 |
| **8** | **R** | **<** | **SAS2197** | **<** | **SAS2498 putative NacetylmuramoylLalanine amidase** | 2 | 2 | 3 | 2 |  |  |  |  |  |  |  |  |  |  |  |
| **9** | **R** | **<** | **SAB2157c butyrylCoA dehydrogenaselike protein** | **<** | **SAB2159c urea transporter** |  |  |  | 1 |  |  |  |  |  |  |  |  |  |  |  |
| **10** | **D** | **>** | **SAS2156 accessory regulator Alike protein** | **<** | **moaA molybdenum cofactor biosynthesis protein** | 2 | 2 | 2 | 1 | 2 | 2 | 2 | 2 | 2 | 2 | 4 | 4 | 4 | 4 | 4 |
| **11** | **D** | **>** | **SaurJH1_2324 sugar transport family protein** | **<** | **SaurJH1_2325** |  |  |  |  | 2 | 2 | 1 | 2 | 2 | 2 |  |  |  |  |  |
| **12** | **R** | **<** | **SAS2004** | **<** | **atpC** | 1 | 1 | 4 |  | 1 | 1 | 1 | 1 | 1 | 1 | 1 | 1 | 1 | 1 | 1 |
| **13** | **R** | **<** | **SAR2172** | **<** | **SAR2173** |  |  | 1 |  |  |  |  |  |  |  | 1 | 1 | 1 | 1 | 1 |
| **14** | **R** | **>** | **SAR2135** | **<** | **SAR2136 DNA binding/iron metalloprotein** |  |  | 1 | 3 |  |  |  |  |  |  |  |  |  |  |  |
| **15** | **D** | **>** | **SAS1939** | **>** | **SAS1940 putative carbonnitrogen hydrolase** | 1 | 1 | 1 |  | 1 | 1 | 1 | 1 | 1 | 1 | 1 | 1 | 1 | 1 | 1 |
| **16** | **D** | **>** | **SAR2109 succinyldiamipimelate desuccinylase** | **>** | **SAR2111 sodium transport protein** |  |  | 2 | 2* | 3 | 3 | 2 | 3 | 3 | 3 |  |  |  |  |  |
| **17** | **R** | **>** | **SAB1874 betahemolysin** | **<** | **SAB1874c leukocidin F subunit** |  |  |  | 1 |  |  |  |  |  |  |  |  |  |  |  |
| **18** | **D** | **<** | **SAB1870c putative GntR family transcriptional regulator** | **>** | **SAB1872** |  |  |  | 1 |  |  |  |  |  |  |  |  |  |  |  |
| **19** | **D** | **<** | **putative lipid kinase** | **<** | **gatB aspartyl/glutamyltRNA amidotransferase subunit B** | 1 | 1 | 3 | 1 | 2 | 2 | 2 | 2 | 2 | 2 | 2 | 2 | 2 | 2 | 2 |
| **20** | **D** | **>** | **SAS1811** | **>** | **SAS1812** | 3 | 3 |  | 2 | 1 | 1 | 1 | 1 | 1 | 1 | 2 | 2 | 4 | 3 | 3 |
| **21** | **D** | **<** | **methionine amipeptidase** | **>** | **SAS1811** | 3 | 3 | 5 | 1 | 2 | 2 | 3 | 3 | 3 | 3 | 3 | 3 | 3 | 3 | 3 |
| **22** | **R** | **>** | **SAS1775** | **<** | **SAS1777** | 1 | 1 | 1 |  |  |  |  |  |  |  | 1 |  | 1 | 1 | 1 |
| **23** | **D** | **>** | **SAB1632 arsenate reductase** | **<** | **SAB1633c** |  |  |  | 2 |  |  |  |  |  |  |  |  |  |  |  |
| **24** | **R** | **<** | **dipeptidase PepV** | **<** | **SAS1678** | 1 | 1 |  | 1 | 1 | 1 | 3 | 3 | 3 | 3 | 7 | 6 | 4 | 4 | 7 |
| **25** | **R** | **<** | **SAR1863** | **<** | **SAR1864 transaldolase** |  |  | 1 |  |  |  |  |  |  |  |  |  |  |  |  |
| **26** | **R** | **>** | **SAB1564** | **<** | **SAB1566c metaldependent hydrolase** |  |  |  | 2 |  |  |  |  |  |  |  |  |  |  |  |
| **27** | **R** | **<** | **SAS1613 putative phosphoesterase** | **<** | **SAS1632 putative DNA binding protein** | 3 | 3 | 2 | 1 | 2 | 2 |  | 2 | 2 | 2 | 1 | 1 | 1 | 1 | 1 |
| **28** | **D** | **<** | **SAS1556** | **<** | **mnmA tRNAspecific 2thiouridylase** | 3 | 3 | 1 | 1 | 4 | 4 | 4 | 4 | 5 | 4 |  |  |  |  |  |
| **29** | **R** | **<** | **SAS1538 putative enterotoxin** | **<** | **SAS1539** | 1 | 1 |  |  | 2 | 2 | 2 | 2 | 2 | 2 |  |  |  |  |  |
| **30** | **D** | **<** | **coproporphyrigen III oxidase** | **<** | **LepA GTPbinding protein** | 2 | 3 | 1 | 2 | 2 | 2 | 2 | 2 | 2 | 2 | 2 | 2 | 2 | 2 | 2 |
| **31** | **R** | **>** | **SAS1385** | **<** | **SAS1386** | 1 | 2 |  |  |  |  |  |  |  |  |  |  |  |  |  |
| **32** | **D** | **>** | **SAS1375** | **>** | **EsbB cell wall enzyme** | 3 | 3 |  |  | 3 | 3 | 1 | 1 | 1 | 1 | 2 | 2 | 2 | 2 | 2 |
| **33** | **D** | **<** | **sucA 2oxoglutarate dehydrogenase** | **<** | **arlS sensor kinase protein** |  |  | 1 |  |  |  |  |  |  |  |  |  |  |  |  |
| **34** | **D** | **<** | **SAS1330 phosphate binding lipoprotein** | **>** | **SAS1331** | 1 | 1 |  |  | 1 | 1 | 1 | 1 | 1 | 1 | 1 | 1 | 1 | 1 | 1 |
| **35** | **R** | **>** | **SAS1288 glycine betaine transporter 1** | **>** | **aconitate hydratase** | 3 | 2 |  |  | 4 | 4 | 4 | 4 | 4 | 4 | 3 | 3 | 3 | 3 | 3 |
| **36** | **D** | **<** | **arlS two component response regulator** | **<** | **SAB1272c** |  |  |  | 1 |  |  |  |  |  |  |  |  |  |  |  |
| **37** | **D** | **>** | **pgsA phosphatidylglycerophosphate synthase** | **>** | **cinA competencedamage inducible protein** |  |  |  | 2 |  |  |  |  |  |  |  |  |  |  |  |
| **38** | **R** | **>** | **rplS 50S ribosomal protein L19** | **<** | **SAS1176** | 2 | 4 |  | 2 |  |  |  |  |  |  |  |  |  |  |  |
| **39** | **D** | **>** | **SAS1128 glyoxalase/bleomycin resistance protein** | **>** | **lspA lipoprotein signal peptide** | 1 | 1 |  |  | 1 | 1 | 1 | 1 | 1 | 1 | 1 | 1 | 1 | 1 | 1 |
| **40** | **R** | **>** | **SAR1138 putative transposase** | **<** | **SAR1139 superantigen like protein** |  |  | 2 |  |  |  |  |  |  |  |  |  |  |  |  |
| **41** | **R** | **>** | **SAS1098** | **<** | **SAS1099 superantigen like protein** | 2 | 4 |  | 1 | 3 | 3 | 3 | 3 | 3 | 3 | 4 | 2 | 2 | 2 | 4 |
| **42** | **R** | **>** | **SAS1092 fibrigenbinding protein** | **<** | **SAS1094** | 4 | 4 |  |  | 4 | 4 | 5 | 4 | 4 | 4 | 5 | 5 | 2 | 2 | 5 |
| **43** | **D** | **>** | **SAS1023 TrkA potassium uptake family protein** | **<** | **SAS1024** | 2 | 2 | 2 |  |  |  |  |  |  |  | 2 | 2 | 2 | 2 | 2 |
| **44** | **D** | **<** | **SAS0997** | **<** | **folD bifunctional 5,10methylenetetrahydrofolate de/cyclo-hydrogenase** | 2 | 2 | 4 | 3 | 2 | 2 | 2 | 2 | 2 | 2 | 2 | 2 | 2 | 2 | 2 |
| **45** | **D** | **<** | **SAR0936** | **>** | **SAR0937** |  |  | 1 |  |  |  |  |  |  |  |  |  |  |  |  |
| **46** | **R** | **>** | **gluD NADspecific glutamate dehydrogenase** | **<** | **SAS0829 GlpQ glycerophosphoryl diester phosphodiesterase** | 2 | 2 | 1 | 2 | 2 | 2 | 2 | 1 | 1 | 1 | 3 | 3 | 3 | 3 | 3 |
| **47** | **R** | **>** | **SAR0872 lipoprotein** | **>** | **SAR0874** |  |  | 3 |  |  |  |  |  |  |  |  |  |  |  |  |
| **48** | **D** | **<** | **SAS0775** | **>** | **SAS0776 TOPRIM domaincontaining protein** | 4 | 4 | 2** | 2** | 4 | 4 | 4 | 4 | 4 | 4 | 4 | 4 | 4 | 4 | 4 |
| **49** | **D** | **>** | **SAS0736** | **>** | **gapR** | 2 | 1 |  |  | 3 | 3 | 4 | 6 | 6 | 6 | 3 | 1 | 1 | 3 | 3 |
| **50** | **D** | **>** | **SAS0729 trxB** | **>** | **SAS0730** | 3 | 3 | 3 | 4 | 5 | 5 | 3 | 6 | 6 | 6 | 2 | 7 | 5 | 5 | 5 |
| **51** | **D** | **>** | **uvrA** | **>** | **hprK** | 2 | 2 | 1 | 1 | 3 | 3 | 3 | 3 | 3 | 3 | 2 | 3 | 3 | 3 | 3 |
| **52** | **D** | **>** | **SAB707 putative transposase** | **>** | **SAB708** |  |  |  | 1 |  |  |  |  |  |  |  |  |  |  |  |
| **53** | **D** | **>** | **prfB peptide chain release factor 2** | **>** | **SAR0809** |  |  | 2 |  |  |  |  |  |  |  |  |  |  |  |  |
| **54** | **R** | **>** | **nrdF ribonucleotide-diphosphate reductase subunit beta** | **>** | **SAS0698 FecCD transport family protein** | 4 | 4 |  | 2 |  |  |  |  |  |  | 4 | 4 | 4 | 4 | 4 |
| **55** | **R** | **>** | **SAS0690** | **<** | **SAS0691 putative diacylglycerol kinase protein** | 3 | 3 | 2 | 2 | 4 | 4 | 4 | 3 | 3 | 4 | 3 | 2 | 2 | 2 | 3 |
| **56** | **D** | **>** | **hicC histidilphosphate amitransferase** | **>** | **SaurJH1_0765** |  |  |  |  | 1 | 1 | 1 | 1 | 1 | 1 | 1 | 1 | 1 | 1 | 1 |
| **57** | **R** | **>** | **SaurJH1_0636 aldo/keto reductase** | **>** | **SaurJH1_0639** |  |  |  |  | 2 | 2 | 2 | 2 | 2 | 2 |  |  |  |  |  |
| **58** | **R** | **>** | **ftsH cell division protein** | **>** | **hslO Hsp33 like chaperonin** |  |  |  | 1 |  |  |  |  |  |  |  |  |  |  |  |
| **59** | **D** | **<** | **sel enterotoxin L** | **<** | **SAB365c** |  |  |  | 1 |  |  |  |  |  |  |  |  |  |  |  |
| **60** | **R** | **>** | **argS arginyltRNA synthase** | **>** | **SAS0576 endonuclease II** | 1 | 1 |  |  |  |  |  |  |  |  | 1 | 1 | 1 | 1 | 1 |
| **61** | **R** | **<** | **SAS0360 putative sodium:dicarboxylate symporter** | **<** | **SAS0361** | 1 | 1 |  |  |  |  |  |  |  |  |  |  |  |  |  |
| **62** | **R** | **<** | **SAS0317** | **>** | **SAS0318 putative acetyltransferase** | 1 | 1 | 1 | 1 | 3 | 3 | 3 | 3 | 3 | 3 | 4 | 3 | 4 | 4 | 4 |
| **63** | **D** | **>** | **SAR0248** | **>** | **SAR0251 putative teichoic acid biosynthesis protein** |  |  | 2 |  |  |  |  |  |  |  |  |  |  |  |  |
| **64** | **R** | **>** | **bglA 6phosphobetaglucosidase** | **<** | **SAS0244** | 3 | 4 | 1** | 1 |  |  | 2 | 2 | 2 | 2 | 1 |  | 1 | 1 | 1 |
| **65** | **D** | **<** | **SAS0230** | **>** | **SAS0231 putative teichoic acid biosynthesis protein** | 4 | 4 |  |  |  |  |  |  |  |  |  |  |  |  |  |
| **66** | **R** | **>** | **SAS0143 putative cation efflux system protein** | **<** | **SAS0145** | 2 | 2 |  |  | 1 | 1 | 3 | 2 | 2 | 2 |  |  |  |  |  |
| **67** | **D** | **<** | **SaurJH1_0072** | **<** | **SaurJH1_0073** |  |  |  |  | 1 | 1 | 1 | 1 | 1 | 1 |  |  |  |  |  |
| **68** | **D** | **>** | **SAB154 maltose/maltodextrin transport system protein** | **>** | **SAB156 NADHdependent dehydrogenase** |  |  |  | 1 |  |  |  |  |  |  |  |  |  |  |  |
| **69** | **R** | **>** | **SAS0121** | **>** | **SAS0122** | 2 | 2 |  |  |  |  |  |  |  |  | 2 | 2 | 2 | 2 | 2 |
| **70** | **D** | **<** | **SAB27c** | **<** | **SAB28c** |  |  |  | 2 |  |  |  |  |  |  |  |  |  |  |  |
| **71** | **D** | **>** | **purA adenylosuccinate synthetase** | **>** | **yycf two component response regulator** |  |  |  | 2 |  |  |  |  |  |  |  |  |  |  |  |
|  |  |  |  |  | **Total (out of 71 loci)** | **39** | **39** | **32** | **36** | **33** | **33** | **33** | **34** | **34** | **34** | **34** | **32** | **34** | **34** | **34** |

**Bibliography**

34. Enright MC, Day NP, Davies CE, Peacock SJ, Spratt BG: **Multilocus sequence typing for characterization of methicillin-resistant and methicillin-susceptible clones of Staphylococcus aureus.** *Journal of clinical microbiology* 2000, **38**:1008-15.

35. Horsburgh M, Clements M, Crossley H, Ingham E, Foster SJ: **PerR Controls Oxidative Stress Resistance and Iron Storage Proteins and Is Required for Virulence in Staphylococcus aureus**. *Infection and Immunity* 2001, **69**:3744-3754.

36. Duthie ES, Lorenz LL: **Staphylococcal coagulase; mode of action and antigenicity.** *Journal of General Microbiology* 1952, **6**:95-107.

37. Anderson J: **Experimental staphylococcal mastitis in the mouse: The effect of inoculating different strains into separate glands of the same mouse**. *Journal of Comparative Pathology* 1974, **84**:103-111.

38. Holden MTG, Feil EJ, Lindsay J a, Peacock SJ, Day NPJ, Enright MC, Foster TJ, Moore CE, Hurst L, Atkin R, Barron A, Bason N, Bentley SD, Chillingworth C, Chillingworth T, Churcher C, Clark L, Corton C, Cronin A, Doggett J, Dowd L, Feltwell T, Hance Z, Harris B, Hauser H, Holroyd S, Jagels K, James KD, Lennard N, Line A, Mayes R, Moule S, Mungall K, Ormond D, Quail M a, Rabbinowitsch E, Rutherford K, Sanders M, Sharp S, Simmonds M, Stevens K, Whitehead S, Barrell BG, Spratt BG, Parkhill J: **Complete genomes of two clinical Staphylococcus aureus strains: Evidence for the rapid evolution of virulence and drug resistance**. *Proceedings of the National Academy of Sciences of the United States of America* 2004, **101**:9786-9791.

39. Moore PLC, Lindsay JA: **Molecular characterisation of the dominant UK methicillin-resistant Staphylococcus aureus strains, EMRSA-15 and EMRSA-16.** *Journal of Medical Microbiology* 2002, **51**:516-521.

40. Kuroda M, Ohta T, Uchiyama I, Baba T, Yuzawa H, Kobayashi I, Cui L, Oguchi a, Aoki K, Nagai Y, Lian J, Ito T, Kanamori M, Matsumaru H, Maruyama a, Murakami H, Hosoyama a, Mizutani-Ui Y, Takahashi NK, Sawano T, Inoue R, Kaito C, Sekimizu K, Hirakawa H, Kuhara S, Goto S, Yabuzaki J, Kanehisa M, Yamashita a, Oshima K, Furuya K, Yoshino C, Shiba T, Hattori M, Ogasawara N, Hayashi H, Hiramatsu K: **Whole genome sequencing of meticillin-resistant Staphylococcus aureus.** *Lancet* 2001, **357**:1225-40.

41. Herron LL, Chakravarty R, Dwan C, Fitzgerald JR, Musser JM, Retzel E, Kapur V: **Genome sequence survey identifies unique sequences and key virulence genes with unusual rates of amino acid substitution in bovine Staphylococcus aureus**. *Infection and immunity* 2002, **70**:3978-3981.

42. Sung JM-L, Lloyd DH, Lindsay JA: **Staphylococcus aureus host specificity: comparative genomics of human versus animal isolates by multi-strain microarray.** *Microbiology* 2008, **154**:1949-1959.
